# Unraveling the IGF System Interactome in Sarcomas Exploits Novel Therapeutic Options

**DOI:** 10.3390/cells10082075

**Published:** 2021-08-13

**Authors:** Caterina Mancarella, Andrea Morrione, Katia Scotlandi

**Affiliations:** 1 Laboratory of Experimental Oncology, IRCCS Istituto Ortopedico Rizzoli, 40136 Bologna, Italy; 2 Department of Biology, Sbarro Institute for Cancer Research and Molecular Medicine and Center for Biotechnology, College of Science and Technology, Temple University, Philadelphia, PA 19122, USA; Andrea.Morrione@temple.edu

**Keywords:** IGF system, sarcomas, IGF inhibitors, Ephrin receptors, Hippo pathway, BET proteins, CXCR4 signaling, combined treatments

## Abstract

Aberrant bioactivity of the insulin-like growth factor (IGF) system results in the development and progression of several pathologic conditions including cancer. Preclinical studies have shown promising anti-cancer therapeutic potentials for anti-IGF targeted therapies. However, a clear but limited clinical benefit was observed only in a minority of patients with sarcomas. The molecular complexity of the IGF system, which comprises multiple regulators and interactions with other cancer-related pathways, poses a major limitation in the use of anti-IGF agents and supports the need of combinatorial therapeutic strategies to better tackle this axis. In this review, we will initially highlight multiple mechanisms underlying IGF dysregulation in cancer and then focus on the impact of the IGF system and its complexity in sarcoma development and progression as well as response to anti-IGF therapies. We will also discuss the role of Ephrin receptors, Hippo pathway, BET proteins and CXCR4 signaling, as mediators of sarcoma malignancy and relevant *interactors* with the IGF system in tumor cells. A deeper understanding of these molecular interactions might provide the rationale for novel and more effective therapeutic combinations to treat sarcomas.

## 1. Introduction

The IGF system regulates a variety of physiological processes including aging, glucose metabolism, growth, and differentiation [1,2,3]. Alterations in IGF equilibrium result in different pathologies including endocrine disorders, skin diseases and cancer [3,4]. Accordingly, multiple anti-IGF targeted therapies were developed and tested in different tumor types [5] but, in spite of encouraging preclinical results, clinical studies have been largely disappointing [6]. Excellent reviews have recently discussed patients’ response to IGF inhibitors [5,7,8]. Notably, among solid tumors, a few but significant achievements were obtained in sarcomas, particularly in Ewing sarcoma [5,6,8,9]. Major factors limiting the efficacy of anti-IGF1R therapy in the clinic include (i.) the lack of validated biomarkers of response for patient selection; (ii.) the development of primary and/or acquired resistance, with absence of significant activity of anti-IGF agents particularly as monotherapy; (iii.) underestimation of the molecular complexity surrounding the IGF axis [6,9]. The variety of biological responses elicited by the IGFs is not solely depending on its canonical components but it also relies on functional cross-talks integrating signals from other factors [2]. The IGF system is finely tuned by a variety of growth factors and hormones [2], post-transcriptional regulators like RNA-binding proteins and non-coding RNAs [9,10,11,12]. The IGF system also interacts with other pathways, including EGFR, HER2, cMET, ALK, or functional partners, like DDR1, E-cadherin, decorin, leading to signaling redundancy or compensatory activity [6,9,12]. To date, combinatorial therapy with anti-IGFs agents represents an attractive therapeutic option. This review will discuss IGFs action in sarcomas and focus on recently discovered mediators of sarcoma malignancy cooperating with the IGF system. We will also discuss the role that these molecular interactions play in tumor development and progression, and the mechanistic rationale for novel combination therapies.

## 2. The IGF System: Major Components and Signal Transduction

The IGF system includes multiple membrane receptors (IGF1 receptor (IGF1R), the insulin receptor (IR), the mannose-6-phosphate receptor (M6P/IGF2R), and the insulin receptor-related receptor (IRR)), three ligands (IGF1, IGF2, insulin), and six circulating IGF binding proteins (IGFBPs) [2,4,13,14]. The IGF1R is a transmembrane tyrosine-kinase receptor (RTK) expressed in most tissue types, including cancer cells, and responsible for long-term actions on growth and development [15]. In its mature form, the IGF1R is constituted by two α chains and two β chains forming an heterotetrameric complex [16,17]. The IGF1R shares a high degree of sequence homology (60%) with the IR [17]. The IR is expressed in two isoforms: the IR-A, generated by alternative splicing of exon 11 and predominantly expressed in fetal tissues and tumor cells, where it mediates mitogenic effects, and the IR-B, which functions in postnatal life in insulin-target tissues like adipose tissue, muscles and liver, where it regulates glucose metabolism [18]. Frequent co-expression of the IGF1R and IR causes the formation of hybrid receptors (HRs) composed by heterodimers of IGF1R and IR-A or IR-B. IGF1R/IR-A and IGF1R/IR-B hybrids have different ligand affinities and elicit different biological responses but the molecular details of hybrids action is not fully characterized [19,20]. Recent findings indicate that HR-mediated effects are more similar to IGF1R than IR, by inducing proliferation and promoting glucose uptake [21]. Still, this study did not differentiate between IR-A and IR-B hybrids [21].

Downstream responses elicited by each receptor strongly depend on specific ligand affinities. The IGF1R shows the highest affinity for IGF1 > IGF2 >> insulin. The IR-A displays the highest affinity for insulin > IGF2 >> IGF1 while the IR-B displays the highest affinity for insulin >> IGF2 > IGF1 [2]. Notably, IGF1R/IR-B hybrids have affinity only for IGF1, while IGF1R/IR-A hybrids have high affinity not only for IGF1 but also for IGF2 and insulin [19].

The other two receptors of the family are the IGF2R and IRR. The IGF2R is a type-1 transmembrane glycoprotein which lacks intrinsic kinase activity and acts by scavenging extracellular IGF2, thus titrating IGF2 levels and suppressing IGF signaling [22]. On the other hand, little is known about the IRR, despite the close homology with the IGF1R and IR. No peptide or protein agonist have been so far identified but recent studies demonstrated that the IRR is an alkaline extracellular pH sensor with a role in the regulation of the acid–base balance [13,23,24].

The three ligands of the system have high homology, with 67% identity between IGF1 and IGF2 and 45–52% sequence homology between IGF1/2 and insulin. However, they are regulated by distinct secretion mechanisms and elicit different properties [25]. Insulin is synthesized by β cells of pancreatic islets in the pancreas, and it is secreted in response to high circulating levels of metabolites like glucose and amino acids. Insulin elicits endocrine short-term actions modulating glucose and amino acids uptake and glucose utilization/production [25]. IGF1 and IGF2 are generated by multiple cell types, but mainly by the liver, mediating endocrine and paracrine/autocrine long-term effects on cell growth, differentiation and survival [25]. Secretion of IGF1 and, to a lesser extent IGF2, is under the control of growth hormone (GH) action. IGF2 secretion mainly depends on genetic imprinting, and it is expressed by the paternal chromosome under the control of the differentially methylated region (DMR) [26]. In addition, the family of RNA-binding proteins insulin-like growth factor 2 mRNA-binding proteins (IGF2BP1, 2, 3) regulates *IGF2* transcript stability at post transcriptional level by modulating IGF2 production in embryos and tumor cells [10]. Importantly, circulating IGF1 and IGF2 are present in inactive complexes with IGFBPs [4]. IGFBPs (IGFBP1-6) are a family of extracellular binding proteins and represent the primary bioregulators of the IGF system [27]. IGFBPs elicit IGF-dependent functions by acting as transport proteins, prolonging the half-life of IGFs, regulating IGFs clearance, providing a tissue specific localization, and modulating IGFs binding to cognate receptors [12,28]. In addition, IGFBPs elicit IGF-independent functions by interacting with different non-IGF proteins at the plasma membrane, in the cytoplasm and in the nucleus, thus playing essential roles in cell proliferation, cell survival, DNA repair, and migration [29,30]. Notably, a very recent finding indicates that IGFBP3 may directly interact with histone 3, suggesting a putative role of IGFBP3 in chromatin remodeling [29].

Ligand binding to the extracellular subunits of IGF1R, IR, or HR induces tyrosine transphosphorylation on the intracellular tyrosine kinase domain [16,17]. Phosphorylated residues act as docking sites for adaptor proteins, such as the family of insulin receptor substrates (IRS) 1-6, Shc and Grb proteins. Phosphorylation of these proteins ultimately activates downstream signaling pathways including the phosphatidylinositol 3-kinase (PI3K), and the mitogen-activated protein kinases (MAPK) pathways [8,31,32]. Upon IRS1/2 activation, PI3K phosphorylates phosphatidylinositol-(4,5)-bisphosphate (PIP2) into phosphatidylinositol-(3,4,5)-trisphosphate (PIP3), which activates downstream substrates including Akt. In turn, Akt regulates glucose metabolism, protein synthesis, cell proliferation and apoptosis via phosphorylation and subsequent activation/inactivation of a variety of substrates including Glut, GSK3, FOXO, TSC1/2, Bad. In parallel, upon IGF1R activation, phosphorylated Shc binds the Grb2/SOS complex determining the downstream activation of the Ras/Raf/MEK/ERK cascade. Translocation of ERK to the nucleus activates a transcriptional program modulating apoptosis, cell proliferation and differentiation. Interestingly, among Grb adapter proteins, Grb10 represents a direct interacting partner for the IGF1R and regulates receptor ubiquitination and stability. In particular, upon ligand stimulation, Grb10 binds the IGF1R connecting the receptor to the ubiquitin protein ligase Nedd4 [33]. The Grb10/Nedd4 complex leads to ligand-induced and Nedd4-mediated ubiquitination and degradation of the IGF1R, reducing IGF1R intracellular signals [33].

In addition, IGF1R can directly phosphorylate the Janus kinases (JAK)-1 and -2, which are involved in cytokine-mediated signaling [34,35,36]. Phosphorylation of JAK proteins leads to the activation of STAT proteins. Of those, STAT3 represents a major mediator of IGF1R transforming potential [36].

## 3. The IGF System in Cancer: A Complex Network of Interactions

The IGF system is involved in the onset and progression of hematopoietic, mesenchymal and epithelial tumors [9,37,38]. Epidemiological studies documented the association between dysregulated IGF action and risk of cancer development. Elevated serum IGF1 levels are associated with increased risk, particularly in thyroid, colorectal, breast and prostate cancer [39]. Patients affected by Laron syndrome, which is characterized by a congenital IGF1 deficiency due to mutation or deletion of the growth hormone releasing hormone (*GH-R*) gene, are protected from cancer development [40]. On the opposite side, bone and bladder cancers incidence appears higher in children in whom a noncancer diagnosis led to GH treatment [41]. In addition, cancer risk is increased in patients with diabetes and obesity, two conditions characterized by hyperinsulinemia [42]. In epidemiological studies, there is no clear association between IGF2 serum levels and cancer risk [43,44]. However, IGF2 is commonly upregulated in cancer and secreted by a broad range of tumor cell types [45,46,47,48]. Notably, IGF2 systemic effects modulate the syndrome known as non-islet cell tumor hypoglycemia [47,48]. While IGF2-linked hypoglycemia itself is not a predictor of tumor risk or aggressiveness, it might play a role in various tumor types [48]. In addition, the term IGF2-omas is currently used to identify those aggressive tumors, secreting IGF2, and characterized by autocrine/paracrine/endocrine effects mediated by the secreted ligand [47,48].

Collectively, the IGF axis modulates major cancer hallmarks [49] including cell proliferation, migration, epithelial-to-mesenchymal transition (EMT), drug resistance, and metabolic reprogramming [8,12,50,51].

The evidence that various cellular and viral oncogenes require an active IGF1R for neoplastic transformation [52] supports the notion that IGF1R expression represents a necessary but not sufficient condition for cell transformation. Similarly, over-expression of the IR-A in NIH 3T3 fibroblasts or immortalized human breast epithelial cells induces a ligand-dependent transformed phenotype but these cells did not induce tumor in nude mice, suggesting that further alterations are required for a complete transformation [53]. Transgenic overexpression of IGF2 increases the risk of developing lung and breast tumors in mice [54,55] while transgenic mice homozygous for a disruption of the Igf2 gene develop tumors with reduced malignancy [56]. Still, IGF2 alone is insufficient for tumorigenesis [44]. The study by Rogler and colleagues demonstrated that transgenic mice overexpressing IGF2 develop a broad spectrum of tumors, including lymphomas, hepatocellular carcinoma, sarcoma, squamous cell carcinoma, and thyroid carcinoma. However, the multiplicity of tumor types and the long tumor latency suggest that IGF2 may act primarily as a tumor progression factor [57].

Dysregulation of IGF axis rarely represents the driver mutation in tumor cells but it rather occurs in association with other events that affect its expression or function [4]. Accordingly, mutations of receptors, ligands, and binding proteins are uncommon. Amplification of the *IGF1R* gene, and its association with oncogenesis and cell survival, has been discovered in subsets of osteosarcoma, a percentage of breast tumors, melanoma, pancreatic adenocarcinoma and gastrointestinal stromal tumors [58,59,60,61,62]. Recently, the novel fusion gene *FN1-IGF1R*, which has strong promoter activity for the kinase domain of IGF1R, has been identified in a subset of inflammatory myofibroblastic tumors [63]. Amplification of *IGF1* and frameshift indel in *IGFBP5* genes has been identified in osteosarcoma (please refer to [12]). Loss-of-heterozigosity of *IGF2R* is present in various tumors (please refer to [12]) and favors IGF2 expression. In vivo results demonstrated that the expression of the Igf2r transgene delays breast tumor onset and decreases tumor multiplicity in Igf2 transgenic mice [64]. Epigenetic modifications cause IGF2 over production in tumor cells. DNA methylation-dependent and -independent mechanisms determine the loss of *IGF2* imprinting (LOI), leading to biallelic *IGF2* expression and enhanced bloodstream secretion levels in Wilms’ tumors and other tumor types including hepatoblastoma, glioma, testicular, colorectal cancers and others, driving malignant processes (please refer to [43,46]). A recent report in colorectal cells indicates that *IGF2* LOI might favor cancer stem cells pluripotency by promoting autophagy, through IGF2-dependent activation of the overexpressed IR-A [65].

Alterations of the IGF axis often depend on aberrant expression or activity of transcription factors. Overexpression of HMGA1 and Sp1 drives enhanced expression of the *IGF1R* and *IR* in tumor cells [66,67,68]. Chromosomal rearrangements leading to oncogenic fusion like *TMPRSS2-ERG*, *EWS-WT1*, and *PAX3-FKHR*, sustain *IGF1R* expression in prostate cancer, desmoplastic small cell tumor and alveolar rhabdomyosarcoma, respectively [69,70,71,72], while *EWS-FLI* favors *IGF1* but inhibits *IGFBP3* transcription in Ewing sarcoma [73]. Mutations in tumor suppressor genes, including *WT1*, *BRCA1*, *VHL*, lead to enhanced *IGF1R* gene expression [74,75,76]. *TP53* mutations upregulated *IGF1R* expression, as well as *INSR*, *IGF2* and *IGFBP3* [77,78,79]. Interestingly, there is a complex interaction between p53 and IGF2 in tumor cells. As demonstrated, p53 suppresses *IGF2* transcription, as a potential mechanism for tumorigenesis [80,81]. Accordingly, forced expression of wild-type p53 in multiple cancer cell lines reduced *IGF2* transcription, particularly in cells expressing mutant p53 protein [80,81]. In addition, there is a synthetic lethal interaction between p53 and IGF2 [79]. In vivo studies employing engineered mice with various allelic doses of Tp53 and Igf2 demonstrated that IGF2 LOI promotes tumorigenesis by inactivating p53 [79,82]. Accordingly, biallelic expression of IGF2 reduced the selective pressure and inactivated the remaining p53 allele in Tp53 heterozygous animals [79,82]. This finding strongly suggests that IGF2 targeting will reduce the fitness of p53-deficient tumors [79,82].

Alterations in the IGF system depend also on dysregulated post-transcriptional mechanisms. Aberrant alternative splicing is associated with prevalent expression of the IR-A vis à vis the IR-B in cancer cells [66,83]. For instance, loss of the splicing factor SRSF3 promotes hepatic carcinogenesis by favoring *INSR* exon 11 skipping thereby inducing IR-A expression [84]. As recently demonstrated, the RNA-binding protein CUGBP1 alters the IR-A:IR-B ratio inducing IR-A prevalence and its mitogenic effects in breast cancer cells [85]. In addition, prevalence of an IR-A/IGF2 loop drives acquired resistance to anti-IGF1R therapies [86]. Enhanced translation of *IGF2* and *IGF1R* occurs via the de novo expression of the oncofetal RNA-binding proteins IGF2BPs (IGF2BP1, IGF2BP2, IGF2BP3) in different tumor types [10,12,87,88,89].

Aberrant IGFBPs modulation leads to increased IGFs bioavailability and binding to the IGF1R or the IR-A and contributes to IGF dysregulation in cancer. Abundance of the metalloproteinase ADAM28 in tumor cells contributes to cell proliferation and metastases through the selective proteolysis of IGFBP3 and the consequent enhanced availability of IGF1 [90]. Similarly, overexpression of the pregnancy-associated plasma protein A (PAPP-A), a cell membrane-associated protease which cleaves and inactivates IGFBP4 and 5, induces an increased local concentration of IGFs triggering tumorigenesis, cell invasion, and drug resistance [91,92]. Interestingly, the proteoglycan decorin acts as a tumor suppressor by binding and negatively regulating IGF1, IGF2, insulin, IGF1R and IR-A activity [12,93,94,95].

IGF activity in cancer is also altered by increased expression or constitutive activation of downstream signaling. Alterations in the tumor suppressor phosphatase and tensin homologue (*PTEN*) gene include both loss-of-function (deletion on chromosome 10) and gain-of-function (PTENΔ51) mutations [96]. Loss of PTEN leads to aberrant upregulation of the IGF/PI3K/Akt pathway and downstream mediators including GLUT1, thereby affecting cancer cell metabolism by enhancing glucose consumption [97]. The PTENΔ51 mutation causes a neomorphic phosphatase-independent activity of PTEN, which activates *IGF1* promoters and increases IGF1 production [96]. As demonstrated by a recent pan-cancer study, the PI3K/Akt/mTOR pathway is mutated in most human tumors [98]. In addition, constitutive activation of IRS1 frequently occurs in cancer and it is associated with development and progression of different tumor types [99,100,101].

## 4. Therapeutic Approaches to Block the IGF System

Three major therapeutic approaches have been developed to block the IGF system: (i.) anti-IGF1R monoclonal antibodies (mAbs); (ii.) tyrosine kinase inhibitor small molecules; (iii.) IGFs neutralizing antibodies and ligands TRAP. A major limitation in the use of these agents is still the lack of a clear rationale in patient recruitment, which may have significantly contributed to poor clinical results. Accordingly, most of the studies were performed in unselected patients further demonstrating that identification of predictive biomarkers to guide patients’ selection still represents an urgent need to optimize the use of anti-IGF agents [6].

### 4.1. Anti-IGF1R mAbs

mAbs against the IGF1R not only block the binding of IGFs to IGF1R but also downregulate IGF1R levels by inducing receptor internalization and degradation, thereby inhibiting receptor downstream signaling [86,102,103]. The first anti-IGF1R mAb αIR3 showed efficacy in preclinical studies by inhibiting cancer cells’ growth both in vitro and in vivo [104]. Several mAbs have been subsequently developed and promising preclinical results were obtained in different tumor models with figitumumab (CP-751871) [105], teprotumumab (R1507) [106], cixutumumab (IMC-A12) [107], dalotuzumab (MK-0646) [108], ganitumab (AMG 479) [109], robatumumab (MK-7454) [110]. However, subsequent clinical trials results were disappointing due to the onset of adverse dose-limiting side effects and lack of antitumor activity in most tumor types. Excellent reviews have recently summarized the results of clinical trials using anti-IGF agents [5,7,8].

Of note, hyperglicemia represents the major side effect correlated with the use of IGF1R mAbs [111]. Indeed, in spite of lack of activity against the IR, some mAbs induce ligand-independent internalization and degradation of IGF1R/IR-A and IGF1R/IR-B hybrids, causing insulin resistance [103,111,112]. In addition, IGF1R blockade might also determine the dysregulation of IGF1R/IGF1/GH, which might determine the inhibition of IGF1 hypoglycemic effect and/or a compensatory increase in circulating GH, leading to increased liver glucogenesis and insulin resistance [111].

The compensatory activation of other RTKs is a common mechanism by which tumor cells develop intrinsic or acquired resistance, thus limiting the antitumor effects of these agents. A phase II study evaluating the efficacy and toxicity of figitumumab in patients with squamous cell carcinoma of the head and neck reported a 41% incidence of hyperglycemia and no clinically significant activity of the mAb in patients [111]. Importantly, molecular analysis conducted in these patients indicated that downregulation of IGF1R levels were associated with a concomitant activation of the PI3K/Akt and MAPK pathways due to compensatory upregulation of the epidermal growth factor receptor (EGFR) [111]. Similarly, compensatory activation of the IR-A and IR-B by insulin and IGF2 [86,113], activation of HER3/AKT pathway, or the induction of a mesenchymal–epithelial transition factor (c-MET) all contribute in conferring resistance to anti-IGF1R mAbs [114,115].

Ganitumab and teprotumumab are currently being tested in clinical trials. Importantly, due to overall lack of IGF1R mAbs efficacy as monotherapy, ganitumab is being tested in combination with other drugs including the Src family knase (SFK) inhibitor dasatinib (NCT03041701), the CDK4/6 inhibitor palbociclib (NCT04129151), and metformin (NCT01042379). An ongoing phase I trial is testing the efficacy of microdevice delivery of different compounds, including ganitumab, in sarcomas (NCT04199026). Teprotumumab is being tested in clinical trials in non-malignant disorders [4]. Despite the fact that the clinical development of cixutumumab has been terminated, cixutumumab conjugated with PEGylated maytansine shows promising in vitro results [116]. Interestingly, an ongoing clinical trial (NCT03316638) is currently testing clinical safety and maximum tolerated dose (MTD) of W0101, a unique IGF1R—targeted antibody—drug conjugate, designed to deliver a cytotoxic auristatin derivative to IGF1R overexpressing tumor cells [117]. Preclinical studies demonstrated the antitumor activity of W0101 and the specificity of this compound for IGF1R and the absence of binding to IR, which should limit potential side effects related to insulin pathway [117].

### 4.2. Tyrosine Kinase Inhibitor Small Molecules

Several tyrosine kinase inhibitor (TKI) small molecules were developed to target the kinase activity of the IGF1R. These agents are divided into ATP-competitive and non-ATP-competitive inhibitors, depending on the ability to block the ATP binding cleft. Based on the high sequence homology between the IGF1R and the IR kinase domains, particularly in the ATP binding cleft (100%) [118], ATP-competitive inhibitors including linsitinib (OSI-906) [119] and BMS-754804 [120], inhibit both the IGF1R and the IR. Despite promising preclinical results, indicating in vitro antiproliferative effects and in vivo antitumor efficacy [119,120], disappointing outcomes were obtained in the clinical setting. While inhibiting both the IGF1R and the IR-A might represent an advantage to avoid compensatory mechanisms in tumor cells, blockade of the IR-B compromises glucose metabolism. None of these compounds are currently under evaluation in the clinic.

Non-ATP-competitive inhibitors, such as the cyclolignan picropodophyllin (PPP; AXL1717), interact with the substrate binding site, thus inhibiting IGF1R signaling without affecting IR action [121]. In preclinical studies, PPP inhibited cell growth and induced cell death in different tumor types [121,122]. In clinical trials, AXL1717, an orally available small molecule whose active agent is PPP, displayed a good safety profile but limited anti-tumor activity. In a phase I trial AXL1717 determined prolonged stable disease in 44% of astrocytoma patients and it caused reversible neutropenia as a major side effect [123]. On the other side, a phase II study in non-small cell lung cancer (NSCLC) patients comparing the efficacy of AXL1717 to docetaxel indicated that no treatment was better than the other in treating locally advanced or metastatic NSCLC [124]. Still, the lower incidence of neutropenia in the AXL1717-treated group confirmed the tolerability of this drug, thus warranting further research [124].

### 4.3. IGFs Neutralizing Antibodies and Ligands TRAP

Neutralizing IGFs ligands represents an alternative therapeutic strategy to block the IGF system. MEDI-573 is a fully human monoclonal antibody that neutralizes both IGF1 and IGF2 and inhibits both IGF1R and IR-A downstream signaling without affecting insulin-dependent IR activation [125]. Preclinical results obtained in embryonic fibroblast cell lines overexpressing the IGF1R and IGF1/IGF2 or IR-A demonstrated that MEDI-573 inhibits in vivo growth in xenograft models [125]. Recent preclinical evidence demonstrated that MEDI-573 induces apoptosis and inhibits tumor growth in a subset of colorectal cancer overexpressing IGF2 [126]. In clinical trials, MEDI-573 determined antitumor activity in advanced solid tumors as shown by stable disease (around 33% of patients), and a favorable tolerability profile without affecting metabolic homeostasis [127]. Despite these encouraging clinical results, a recent trial evaluating MEDI-573 efficacy alone or in combination with aromatase inhibitors in a population of hormone-sensitive metastatic breast cancer showed no significant difference in progression-free survival in patients treated with aromatase alone or in combination with MEDI-573 (NCT01446159). While no clinical study is currently ongoing for MEDI-573, active clinical trials are under way for the monoclonal antibody BI 836845 (xentuzumab). BI 836845, a humanized IgG1 monoclonal antibody, binds IGF1 and IGF2, thereby inhibiting downstream signaling essential for survival and tumor growth [128]. In vivo evidence in hepatocellular carcinoma showed efficacy of BI 836845 in xenograft tumors and prolonged survival of treated mice [129]. Similarly, in prostate cancer, xentuzumab + enzalutamide inhibited the growth of castration-resistant patient-derived xenograft (PDX)-derived cells with acquired resistance to enzalutamide and improved in vivo survival [130]. In the clinic, two preliminary studies testing xentuzumab as single agent in advanced solid tumors indicated a good tolerability and preliminary anti-tumor activity [131]. However, a phase Ib/II study indicated that the addition of xentuzumab to everolimus/exemestane did not improve progression free survival in breast cancer patients, leading to early termination of this trial [132]. Still, evidence of progression free survival was observed in patients without visceral metastases when treated with xentuzumab/everolimus/exemestane, leading to initiation of the phase II XENERA™-1 trial (NCT03659136) [132].

Among the IGF-targeting strategies, an emerging area of research is currently focusing on IGF-Trap. The IGF-Trap is a heterotetramer, composed of the extracellular domain of the IGF1R fused to the Fc portion of human IgG1 [133,134]. The IGF-Trap binds IGF1 and IGF2 with higher affinity than insulin, thus avoiding metabolic effects [133,134]. In addition, it inhibits IGF1R-driven cellular functions such as migration, proliferation and survival in various carcinoma cellular models [133,134]. The IGF-Trap significantly reduces metastatic spread of colon and lung carcinoma cells to the liver, representing a novel promising alterative to IGF1R or IGF1/IGF2 antibodies [133,134].

## 5. The IGF System in Sarcomas: Functions and Response to Therapies

Sarcomas represent a rare clinical and molecular heterogeneous group of mesenchymal neoplasms, categorized as bone and soft-tissues tumors [135]. Despite the lack of clear epidemiologic evidence connecting the IGF system to enhanced risk of sarcoma development, a large amount of preclinical data demonstrated a pivotal role of the IGF axis in these tumors. This section will summarize the mechanisms underlying IGF dysregulation and their impact in sarcoma development and progression as well as preclinical and clinical results using anti-IGF agents. Molecular complexity surrounding the IGF system in sarcomas will be discussed throughout this section.

### 5.1. Osteosarcoma

The IGF system is linked to the pathogenesis and progression of osteosarcoma, the most common primary tumor of bone in children and adolescents [135,136]. Early experimental studies demonstrated that human osteogenic sarcoma cells were responsive to IGF1 for mitogenesis while reduction in IGF1 levels by hypophysectomy inhibited the aggressive metastatic behavior of osteosarcoma both in vitro and in vivo [137]. In addition, expression of the IGF1R, IGF1 and IGF2 was detected in most osteosarcoma cell lines and patient-derived tissues [138]. At molecular level, gene expression analysis comparing osteosarcoma with normal bone cells demonstrated the simultaneous loss of all the IGFs inhibitors, such as IGFBPs and IGFBPrPs, as an essential step in the development of this tumor [139]. An active IGF axis in osteosarcoma functionally modulated cell in vitro migration, coloy formation, cell cycle progression, EMT and in vivo xenograft tumor growth [140] as well as resistance to radiation, docetaxel, cisplatin and doxorubicin [141]. Increased IGF1/IGF1R expression has been demostrated in osteosarcoma patients’ tissues and it was associated with a poor prognosis [142,143]. In spite of all this evidence Benini and colleagues showed that treatment of osteosarcoma cell lines with the anti-IGF1R antibody αIR3 was ineffective as single therapy [144]. In fact, the authors showed that autocrine loops mediated by EGFR and NGFR functionally interacted with the IGF axis, thereby significantly limiting the efficacy of solely targeting the IGF1R [144]. Additional preclinical evidence supported the use of anti-IGF agents, including PPP and R1507, in ostesarcoma [145,146]. However, the results of clinical trials evaluating those agents as monotherapy were disappointing, as only 3/60 (5%) cases of osteosarcoma treated with the anti-IGF1R mAb robatumumab showed complete or partial response [147]. Factors that might have contributed to the inefficacy of anti-IGF1R targeted therapy rely on: (i.) the complexity of the osteosarcoma genome and the redundancy of compensatory autocrine loops; (ii.) paucity of osteosarcoma cases characterized by actionable somatic alterations in IGF signaling genes.

Osteosarcoma is characterized by the simultaneous amplification of oncogenes, loss of tumor suppressors and inactivation of DNA-repair genes [60,148]. Accordingly, high expression of various RTKs including c-Met, HER-2, VEGFR-3, IR, and PDGFR-β, IGF2R, and EGFR and activation of the downstream PI3K/Akt pathway, has been demonstrated in osteosarcoma cells [149,150]. In line with the genetic complexity of this tumor, more promising results were obtained in combination studies. Partial responses were registered in 3/24 (12.5%) osteosarcoma cases treated with a combination of anti-IGF1R mAb cixutumumab and the mTOR inhibitor tensirolibus [151]. OncoLAR®, a long-acting somatostatin analog, which reduces IGF1 levels, was well tolerated but lacked clinical response in osteosarcoma patients [152]. Based on the co-expression of IGF1R and HER2, OncoLAR® was tested in combination with tamoxifen (NCT00001436), but there was no tumor response [152].

Whole exome genome analysis in osteosarcoma has recently indicated that actionable somatic alterations in IGF signaling genes actually occur in a small subset of cases. In particular, somatic copy number variations of the IGF gene family occurr in about 7% of cases [60,153] while fluorescence in situ hybridization (FISH) indicated a 9.2% [153] to 14% [60] of osteosarcoma cases displaying amplification of *IGF1R* gene with corresponding increased *IGF1R* transcription and downstream activation of the PI3K/Akt/mTOR pathway [153]. Despite the fact that any therapeutic strategy solely targeting the IGF1R is likely insufficient to prevent the activation of downstream signaling pathways in osteosarcoma, this evidence suggests that a genomic-guided approach for the use of anti-IGF agents in patients carrying *IGF1R* amplification might be considered for future clinical trials.

Other events have been reported to govern IGF1R expression in osteosarcoma, further demonstrating the complexity which surrounds the IGF axis in this tumor. For instance, recent data demonstrated that the mediator of EMT process cysteine-rich protein 61 (CYR61/CCN1), which correlates with osteosarcoma aggressiveness in preclinical model and in patient tumor samples, increased IGF1R and IGF1 expression in osteosarcoma cells, thereby modulating cell–cell interactions and motility [154]. Non-coding RNAs regulate IGF1R in osteosarcoma. For example, miRNA16, which negatively regulates IGF1R/Kras/Raf1/MEK/ERK pathway and cell proliferation, is downregulated in osteosarcoma compared to normal controls [155].

### 5.2. Ewing Sarcoma

Ewing sarcoma is the second most common pediatric bone tumor. It is characterized by a recurrent somatic translocation which fuses *EWSR1* to a member of the ETS family of transcription factors, mainly *FLI1* (85% of cases) [156]. The resulting chimera, EWS-FLI1, acts as an aberrant transcription factor and represents the ocogenic driver of this disease [156]. The IGF system is critical in regulating Ewing sarcoma development and progression via a complex interaction with EWS-FLI1. Original experiments conducted in fibroblasts derived from mouse embryos homozygous for a null mutation of the Igf1r gene (R-cell) or wild type littermates demonstrated that only cells expressing both the IGF1R and EWS-FLI1 form colonies in soft-agar, demonstrating that the IGF1R was required for EWS-FLI1-mediated transformation [73]. Conversely, EWS-FLI1 alters IGF signaling. EWS-FLI1 represses the transcription of *IGFBP3*, while favoring the transcription of *IGF1*, which is a critical step in Ewing sarcoma tumorigenesis [157,158]. As a result, Ewing sarcoma cells are characterized by a unique autocrine loop, mediated by the IGF1R/IGF1 axis, which strongly contributes to cellular malignancy [159]. Pharmacological blockade of the IGF1R using mAbs or TKIs inhibited Ewing sarcoma cells proliferation, soft-agar growth, migration and inhibited downstream signalling, increased apoptosis, and blunted in vivo tumor growth [159,160,161,162]. In the clinical setting, anti-IGF agents as monotherapy showed encouraging anti-tumor activity, being effective in a subset of Ewing sarcoma patients. Several anti-IGF1R mAbs showed promising results. Ganitumab was associated with 11% clinical benefit, 42% disease stabilization and 5% partial response [163]. R1507 gave partial response in 7% of cases, complete response in 2.3% and clinical response in 10% of cases [164]. Figitumumab gave disease stabilization in 23% of cases and partial response in 14% of cases [165] or, in a second study, disease stabilization in 37% of cases [166]. Robatumumab gave disease stabilization in 27% of cases and 7% of partial response [147]. To date, predictive biomarkers to identify the subset of patients are most likely to benefit from anti-IGF1R therapy have not been identified. Expression levels of IGF1R and IGF1 were considered in different studies as biomarkers of response to anti-IGF agents [147,164,165,167]. However, gene signatures associated with response to robatumumab in tissues from Ewing sarcoma patient responders, non-responders and controls, identified genes were associated with mTOR and Akt action but not the IGF1R and IGF1 [147]. In Ewing sarcoma patients treated with figitumumab [165] and R1507 [164] pretreatment serum levels of IGF1 were associated with increased survival but not with drug response. Published data indicated that nuclear localization of the IGF1R, evaluated by immunohistochemistry in different sarcoma specimens including Ewing sarcoma might work as a predictive marker of response to anti-IGF1R mAbs [167]. At the preclinical level, expression levels of the RNA-binding protein IGF2BP3, in combination with the IGF1R/IR ratio, were suggested as putative predictor of response to linsitinib (OSI-906) in Ewing sarcoma cells [162]. Accordingly, Ewing sarcoma cells with decreased expression of IGF2BP3 and the IGF1R, but increased compensatory expression of the IR, displayed higher sensitivity to OSI-906 compared to cells expressing high levels of IGF2BP3 [162].

The lack of response to IGF agents in Ewing sarcoma likely depends on an aquired resistance mechanisms developed by these cells to adapt to IGF1R depletion. Enhanced expression of the IR-A and its mitogenic ligand IGF2 is a major mechanism of resistance to anti-IGF1R therapy in Ewing sarcoma. In resistant cells, an IR-A/IGF2 axis activated upon blockade of canonical IGF1R/IGF1 signaling constitutes an alternative autocrine loop sustaining cell growth via MAPK pathway [86]. Accordingly, linsitinib, which blocks both the IGF1R and IR, was evaluated in Ewing sarcoma patients but no response was evidenced (NCT02546544). Similarly, compensatory up-regulation of IRS-1, PI3K, STAT3 and p38 MAPK emerged as mechanisms of resistance to different IGF1R inhibitors in Ewing sarcoma [168].

Combinatorial approaches with anti-IGF1R inhibitors in association with either other drugs or chemotherapy are currently under investigation in order to avoid pathway crosstalks and acquired resistance. Mechanism-based evidence provided the rationale to combine anti-IGF1R agents with the mTOR inhibitor temsirolimus [169], as in fact mTOR inhibitors activate a feedback loop resulting in Akt activation downstream of the IGF1R [168,169,170]. In clinic, encouraging results were obtained in Ewing sarcoma patients by combining cixutumumab with temsirolimus. A total of 12% of patients achieved complete response and 18% experienced disease stabilization [170]. Other evidence demonstrated that the activation of the IGF1R promoted resistance to cyclin-dependent kinase (CDK)4/6 inhibitors in Ewing sarcoma cells. Accordingly, dual targeting with CDK4/6 and IGF1R inhibitors is currently evaluated in this disease [171]. A combinatorial approach with anti-IGF1R mAb ganitumab and the CDK4/6 inhibitor palbociclib is currently under investigation in Ewing sarcoma (NCT04129151). Finally, the rational for combining anti-IGF1R agents and trabectedin was provided by in vitro studies showing that trabectedin enhanced the binding of EWS-FLI1 to *IGF1R* promoter leading to IGF1R upregulation. Accordingly, combination of trabectedin with anti-IGF1R mAb AVE1642 promoted antitumor activity in Ewing sarcoma xenografts [172].

### 5.3. Rhabdomyosarcoma

Rhabdomyosarcoma is the most common pediatric soft tissue sarcoma of the muscle [173]. Two major histopathological subtypes exist: alveolar and embryonal [173]. The alveolar subtype is characterized by a recurrent t(2;13) translocation genereting a fusion between the *PAX3* and *FKHR* (*FOXO1*) genes. An additional but rare chromosomal translocation t(1;13) results in a fusion between *PAX7* and *FOXO1*. The PAX3/7-FOXO1 fusion protein acts as a more potent transcriptional activator than PAX3/7 alone. Importantly, it drives pathogenesis and is associated with increased aggressiveness and poor prognosis [174]. Dysregulation of the IGF system plays a key role in both subtypes of rhabdomyosarcoma as it is in fact one of the signaling pathways associated with metastases and poor prognosis in patients [175]. Mechanistically, IGF2 is overexpressed in both subtypes of rhabdomyosarcoma due to loss of imprinting or heterozygosity at the 11p15.5 locus [176,177]. There is evidence that the RNA-binding protein IGF2BP2 is highly expressed in embryonal rhabdomyosarcoma cells, where it regulated cell growth and survival [178]. Interestingly, *IGF2* mRNAs are directly regulated by IGF2BP2 in those embryonal cells, thereby suggesting a putative post-transcriptional mechanism of IGF2 dysregulation in this tumor [178]. Importantly, PAX3/7-FOXO1 is a transcriptional regulator of the *IGF1R* and enhances receptor expression [174]. Microarray analysis demonstrated that *IGF2* and *IGF1R* are both highly expressed in rhabdomyosarcoma cell lines, xenografts, and human tumor samples [179]. As demonstrated by Makawita et al., rhabdomyosarcoma therapy-naive biopsies were positive for IGF1R expression in 72% of alveolar subtype and 61% of embryonal subtype [180]. Overall, IGF2 and IGF1R constitute a potent autocrine signalling axis, which promotes proliferation of rhabdomyosarcoma tumors [181]. Accordingly, in vitro and in vivo studies confirmed that inhibiting the IGF axis blocks tumor proliferation and metastasis in rhabdomyosarcoma. For instance, the IGF1R TKI PPP inhibited rhabdomyosarcoma proliferation, chemotaxis and adhesion [181]. It also attenuated MAPK phosphorylation, thereby determining cell cycle arrest in the G2/M phase [181]. In addition, PPP increased sensitivity to chemotherapy, specifically to vincristine and cisplatin [181]. Furthermore, SCID-beige mice injected with alveolar rhabdomyosarcoma RH30 cells and treated with PPP developed smaller tumors and displayed significantly decreased seeding into bone marrow [181]. Anti-IGF1R agents were tested as monotherapy in clinical trials including rhabdomyosarcoma patients. A phase 2 trial with the anti-IGF1R mAb R1507 indicated that the treatment was safe but with limited anti-tumor activity as it in fact demonstrated only 2% of partial response and 16% of disease stabilization [182]. Similar results were obtained in a phase 2 trial testing the safety and efficacy of the IGF1R mAb cixutumumab [183]. Addition of cixutumunab to intensive multiagent chemotherapy did not improve the outcome of rhabdomyosarcoma patients compared to chemotherapy alone [184]. Several studies have later focused on the characterization of the mechanisms of aquired resistance to IGF1R blockade in order to identify combinatorial approaches that will improve the efficacy of IGF agents [179]. Constitutive downregulation of IGFBP2, which inhibits IGF1R phosphorylation and signaling, was identified as a possible mechanism for resistance to IGF1R antibody [185]. It is important to mention that, in addition to the IGF axis, several autocrine and paracrine signaling pathways might contribute to the malignant propensity of rhabdomyosarcoma histologic subtypes inlcuding the cMET, FGFR4 and PDGFR cascades, which all activate Akt signaling [173]. Accordingly, progression of rhabdomyosarcoma tumors and resistance to IGF1R inhibition were associated with reactivation of Akt in spite of suppression of IGF1R action [186]. The combination of the anti-IGF1R mAb h7C10 [112] and the mTOR inhibitor rapamycin significanlty inhibited in vivo xenograft tumor growth [186], preventing tumor escape observed after the anti-IGF1R mAb h7C10 treatment alone and limiting the potential problem of mTOR inhibitors-evoked up-regulation of AKT [186]. The effect of mTOR inhibitors is the block on the feed-back negative mechanisms which cause sustained AKT activation.

In parallel, preclinical studies demonstrated that IGF1R inhibition with either mAbs or TKIs activated YES kinase, a member of the SRC family tyrosine kinases (SFKs), as a resistance pathway in rhabdomyosarcoma cell lines and xenografts [179]. Accordingly, dual blockade of IGF1R and SFKs caused a dramatic inhibition of the Akt pathway, enhanced apoptotic response and inhibited in vitro and in vivo cell growth when compared to single treatment [179]. Interestingly, the combination of the anti-IGF1R mAb ganitumab with the SFKs inhibitor dasatinib is being currently tested in patients affected by rhabdomyosarcoma, both alveolar and embryonal subtypes (NCT03041701). This trial is still recruiting patients and therefore the results are not yet available.

The interruption of IGFs autocrine rhabdomyosarcoma circuits through passive or active immune neutralization of IGF2 has been recently investigated in a preclinical setting. BALB-p53Neu male mice, injected to generate IGF2-dependent rhabdomyosarcoma, were treated with anti-IGFs antibodies and the results indicated a significant delay in the sarcoma onset and prolonged overall survival [186]. Interestingly, mice vaccinated with human *IGF2* DNA, producing antibodies cross-reacting with mouse IGF2, were partially protected from a subsequent injection of RMSp53Neu-5 rhabdomyosarcoma cells, showing a significant 60% decrease in the number of lung metastases when compared to untreated controls [186].

### 5.4. Synovial Sarcoma

Synovial sarcoma is a rare soft tissue malignancy, accounting for 8% of all soft tissue tumors and affecting young adults [187]. The genetic hallmark of synovial sarcoma is the chromosomal translocation t(X;18)(p11;q11), which leads to a fusion of *SS18* (*SYT*), which encodes a SWI–SNF complex component, to one of the homologues *SSX* genes (*SSX1*, *SSX2*, rarely *SSX4*), which encodes instead a transcriptional repressor [188,189]. The resulting chimeric SS18–SSX fusion proteins play a crucial role in the tumorigenesis of synovial sarcoma [190]. However, specific functions and molecular processess elicited by the fusion proteins are still not well-characterized. SS18-SSX alters gene expression at epigenetic level through the association with SWI/SNF and Polycomb chromatin remodeling complexes [188,191]. Among the altered genes, the SS18–SSX fusion protein dysregulates IGF sigalling [192]. Microarray studies demonstrated that the induction of SS18-SSX2 in HEK-FUS cells leads to up-regulation of *IGF2* via altered methylation at the *IGF2* locus [192]. From a functional standpoint, all synovial sarcomas express IGF2. In addition, most cases (around 70%) show an activated IGF1R and the vast majority are characterized by the activation of both the Akt and MAPK pathways [187,193]. The IGF2/IGF1R/Akt-MAPK axis drives synovial sarcoma cells growth and migration. Accordingly, ihibition of IGF1R signalling using the TKI NVP-AEW541-impaired phosphorylation of Akt and MAPK, increased apoptosis, diminished mitotic activity and migration of synovial sarcoma cells [193]. Significantly, SS18–SSX and IGF1R cooperate in synovial sarcoma malignancy, playing important but different roles in maintaining malignant growth of synovial sarcoma cells. In particular, SS18–SSX controlled cell proliferation, while the IGF1R protected from apoptosis [194]. Preclinical studies investigating the effects of IGF blockade in vitro and in vivo synovial sarcoma models gave promising results [195]. In the clinic, a limited number of studies have been conducted so far but the results were not encouraging in phase 2 trials. Evaluation of the anti-IGF1R mAb R1507 efficacy in 23 synovial sarcoma patients showed 4 cases (17%) of disease stabilization as best response [182], while the mAb figitumumab was associated with one patient experiencing disease stabilization among 13 patients with multiple sarcoma subtypes [166].

Overall, the limited efficacy of a therapeutic approach based on a single RTK inhibition is not an unexpacted result. On one side, the complexity surrounding the IGF system in synovial sarcoma might limit the efficacy of anti-IGF agents as monotherapy. For example, synovial sarcoma cell lines and patients all display elevated levels of the IGF1R-interacting partner SRC, which is activated and promotes cell survival and proliferation [196]. In vitro studies demonstrated that SRC activation was induced by the SS18/SSX/IGF2/IGF1R axis and that inhibition of SRC using dasatinib caused the inhibition of IGF1R signalling, supporting the view that this interaction might alter the bioactivity of the IGF system [196]. Dysregulation of IGF1R signaling in synovial sarcoma might also depend on additional mechanisms. For instance, targeting the heparanase/heparan sulfate proteoglycan system using a supersulfated low molecular weight heparin (ssLMWH) inhibited cell colony growth and downregulated the IGF1R in synovial sarcoma preclinical models [195]. Overall, ssLMWH in combination with the anti-IGF1R TKI BMS754807 showed synergistic effects both in vitro and in vivo [195]. On the other hand, multiple RTKs actually contribute to synovial sarcoma cell growth, thus limiting a therapeutic approach based on the inhibition of a single RTK. Indeed, a recent mass spectrometry-based phosphoproteomic profile from a panel of synovial sarcoma cell lines identified a major role of ALK and MET as possible oncogenic drivers of this neoplasia [197].

### 5.5. Desmoplastic Small Round Cell Tumor

Desmoplastic small round cell tumor is a rare primitive soft tissue sarcoma of children and young adults, mostly confined to the abdome [198]. The driver mutation of this patholgy is the recurrent t(11;22)(p13;q12) translocation that fuses the 5′ exons of the *EWS* gene to the 3′ exons of the *WT1* gene [71]. The *EWS-WT1* gene fusion generates a chimeric protein which acts as aberrant transcription factor. Various target genes of EWS-WT1 have been previously described including *c-MYC* and RTKs coding genes like *PDGF*, *FGFR*, *EGFR* and *IGF1R* [71,198]. In the latter case, EWS-WT1 binds the promoter of the *IGF1R* gene, thereby significantly enhancing its transcription. Despite a substantial paucity of in vitro functional studies, the EWS-WT1/IGF1R axis likely constitutes one of the major mechanisms for the etiology and/or progression of desmoplastic small round cell tumor malignancy [199]. Published data indicate that EWS-WT1-mediated upregulation of IGF1R leads to enhanced ligand/receptor interaction and growth stimulation [71,200]. Among other components of the IGF system, two different case reports described abnormal IGF2 secretion in serum of two patients affected by desmoplastic small round cell tumor [201,202]. In those patients, IGF2 levels were associated with tumor hypoglycemia [201,202]. Recent RNAseq confirmed the strong over expression of IGF2 across desmoplastic small round cell tumors and ChIP seq analyses demonstrated that EWS-WT1 direclty targets *IGF2*, contributing to its over expression [203]. Clinical studies testing the safety and the efficacy of anti-IGF agents in desmoplastic small round cell tumor produced encouraging results. The anti-IGF1R ganitumab in 16 patients affected by desmoplastic small round cell tumor was well-tolerated and was associated with partial response in 6% of cases, disease stabilization in 63% of cases and disease stabilization for more than 24 weeks in 3% of cases [163]. In addition, the combination of the anti-IGF1R mAb cixutumumab with the mTOR inhibitor temsirolimus resulted in stable disease for two of three desmoplastic small round cell tumor patients for at least 5 months [170]. Similarly to Ewing sarcoma, the identification of biomarkers predictive of response in patients most likely to benefit from anti-IGF1R therapy remains elusive at this time.

## 6. Perspectives for Novel Therapeutic Combinations in Sarcomas

The IGF system operates in a network of complex interactions that might limit the efficacy of therapeutic agents targeting this axis in sarcomas. A better understanding of these connections might provide the rationale for designing of novel therapeutic combinations to test in the clinic. This section will provide novel perspectives of additional interactions with the IGF system and define their impact on tumor cells. Particularly, we will focus on those *interactors* with a reported role in sarcoma that might work as “druggable” targets in cancer.

### 6.1. Ephrin Receptors

The Ephrin (Eph) receptors represent the largest family of RTKs and, in association with their plasma membrane-bound ligands (Ephrin), regulate cell–cell communication, cell proliferation, migration and cell adhesion [204]. Ehp receptors are single transmembrane proteins composed of an N-terminal extracellular domain, which modulates ligand binding, and the intracellular domain, which holds enzymatic activity [204]. Based on their ligand binding affinity, Eph receptors are grouped into A and B including nine EphA receptors and five EphB receptors in humans. Similarly, ligands include five members of ephrin A family and three members of the ephrin B family. Ligand–receptor interactions are mainly restricted between ligands and receptors within either the A or B family [204]. Ligand binding requires direct cell–cell contact and evokes Eph receptor autophosphorylation and subsequent activation of intracellular effectors like Src family kinases and the Ras/Rho family. Interestingly, the ephrin/Eph receptor interaction leads to bidirectional signals, with forward signaling affecting receptor-expressing cells and reverse signaling affecting ligand-expressing cell. Eph receptors drive crucial physiologial processes, such as tissue development and neural targeting, as well as pathologic conditions, like cancer [204]. Ephrin and Eph receptors are up-regulated in differet tumor types, modulating carcinogenesis, tumor progression and metastasis [204]. The ephrin/Eph receptor axis might elicit pro- or anti-oncogenic functions depending on cellular context, adding a further level of complexity to this system [205]. Several approaches have been developed to target Eph receptors in cancer [206,207,208] and later evaluated in clinical trials. For example, a phase I dose-escalation study of sEphB4-HAS, a soluble fusion protein that binds and sequesters the ligand for EphB4, in combination with chemotherapy, cetuximab and radiotherapy is ongoing in squamous cell carcinoma of head and neck patients (NCT04091867). A phase I trial is testing the safety profile and effective dose of an EphA2 siRNA in patients with solid tumors (NCT01591356). MM-310, a liposomal formulation of a docetaxel prodrug using EphA2 as drug delivery target on cancer cells, has been tested in patients with solid tumors but results are not yet available (NCT03076372).

Functional interactions between the IGF and the Eph axis have been demonstrated in tumor cells. In preclinical breast cancer models, the IGF1/IGF1R/Akt axis sustained mRNA expression of *EphB4*, thereby promoting cell proliferation and migration responses, which were sentitive to the PI3K inhibitor LY294002 [209]. In malignant mesothelioma cells, upon autocrine IGF2 stimulation, IR-A phosphorylates EphB4 at tyrosine 987, thus maintaining EphB4 steady-state levels [47]. Accordingly, neutralization of the IR-A/IGF2 loop using anti-IGF2 antibodies determined EphB4 dephosphorylation and increased receptor ubiquitination and degradation [46,47]. An interesting crosstalk has been described between the PI3K/Akt pathway and EphA2 in tumor cells [210]. Upon growth factor/growth factor receptor activation, Akt phosphorylates EphA2 on serine 897, promoting cell migration in a ligand-independent manner. Of note, stimulation with EphrinA1 leads to dephosphorylation of EphA2 on Ser897 and inhibition of migration [210]. Overall, these results suggest a putative role of the IGF1R in the regulation of the Akt/EphA2 axis, although this hypothesis remains speculative at the moment.

To date, a functional interaction between the IGF system and the Eph axis has not been described in sarcoma models. However, Eph and IGF1 signaling pathways cross-talk to regulate bone and skeletal muscle cells function [211,212] and EphA2 and EphB4 exert oncogenic effects in different types of sarcomas, supporting the rationale for targeting Eph-dependent pathways in those tumors [208,213,214,215,216,217,218,219,220].

In myoblast cell lines, the EphrinA1/EphA axis suppresses Ras-ERK1/2 signaling, thus favoring IGF1-induced myogenesis [212]. EphA4-knockout mice are characterized by low circulating levels of IGF1 and dysregulated growth associated with deficiency in body size and skeletal system [211]. Published data suggest that, in addition to GH, EphA4 might represent an additional component in the modulation of IGF1 levels, as in fact EphA4 forms a complex with the GH receptor, JAK2, and STAT5B, thereby enhancing IGF1 expression [211]. Overall, these results suggest that dysregulation of these functional cross-talks might result in the onset of musculoskeletal tumors.

EphA2 is up-regulated in osteosarcoma, Ewing sarcoma and rhabdomyosarcoma [208,213,214,215,216,217,218,219,220].

Genome-wide microarray and immunohistochemistry studies demonstrated de novo expression of EphA2 in osteosarcoma patients as compared to healthy bone tissues [214,218]. From the functional standpoint, binding of ephrinA1 to EphA2 activates the oncogenic MAPK signaling and stimulates osteosarcoma metastasis [214]. Most Ewing sarcoma patients (90.4%) and cell lines express EphA2 protein. Interestingly, in those cells, EphA2 acts in both ligand-dependent and -independent manners. In the presence of caveolin1, stimulation with ephrinA1 promoted Tyr-phosphorylation of EphA2, Akt activation and promotion of the pro-angiogenic factor bFGF expression and vascularization [220]. Instead, EphA2 promoted Ewing sarcoma migration via ligand-independent phosphorylation at S897 [215]. Moreover, in Ewing sarcoma cells, a feedback loop exists between EphA2 phosphorylation and the MAPK pathway, as in fact silencing of EphA2 decreased both Akt and ERK phosphorylation while EphA2 phosphorylation at S897 was reduced exclusively by MEK inhibitors [215]. EphA2 is up-regulated in embryonal rhadbomyosarcoma primary tissues and cell lines, driving cell proliferation, migration and blocking myogenic differentiation [217]. Accordingly, treatment of embryonal rhadbomyosarcoma cells with GLPG1790, a small molecule that inhibits various Eph receptors, including A2, inhibited cell viability, induced cell cycle growth arrest, inhibited cell migration, promoted myogenic differentition and inhibited tumor growth in xenograft mouse models [217]. In ostesarcoma and Ewing sarcoma preclinical models, EphA2-directed chimeric antigen receptor (CAR) T-cell therapy showed potent in vitro and in vivo anti-tumor efficacy [216].

EphB4 has a role in tumorigenesis and the metastatic process of synovial sarcoma [208] and the alveolar subtype of rhabdomyosarcoma [219]. EphB4 mRNA and protein expression were upregulated in synovial sarcoma patients [208] and in vitro blockade of EphB4 using either siRNA approaches or the specific EphB4 kinase inhibitor NVP-BHG712 inhibited cell proliferation and cell migration [208]. EphB4 and its ligand EphrinB2 are both expressed in the alveolar subtype of rhabdomyosarcoma [219]. In this tumor, EphB4 signaling can be induced by its ligand EphrinB2 [205,219] or by the cross-talk with PDGFRβ, which facilitates PDGF ligand-dependent, ephrin ligand-independent activation of EphB4 [213,219]. In accordance with the complexity which surrounds EphB4, preclinical evidence indicated that inhibition of EphB4 signaling is not a viable monotherapy for alveolar rhabdomyosaroma. Indeed, direct EphB4 inhibition using the humanized inhibitory antibody VasG3 failed to affect tumor growth and survival rates in alveolar rhabdomyosarcoma cells xenograft models [219]. In parallel, inhibition of EphrinB2/EphB4 forward signaling using soluble EphB4, which binds and sequesters the EphrinB2 ligand, resulted in a moderately decreased tumor growth of U48484 cells in an orthotopic allograft [219]. In contrast, the small molecule dasatinib, which inhibits both EphB4 and its partner PDGFRβ, resulted in a significant inhibition of in vitro tumor cell viability as well as decreased in vivo tumor growth and significantly prolonged survival [213].

**Figure 1:**
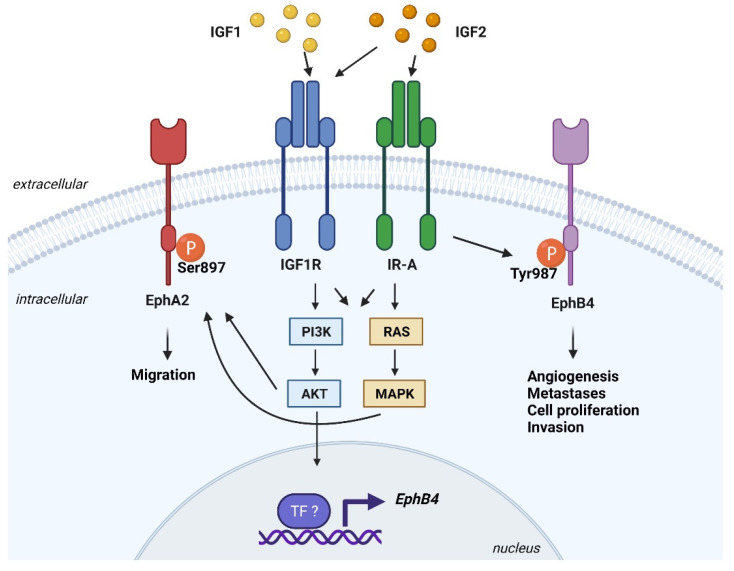
Schematic representation of the functional connections between the IGF system and the Ephrin axis in cancer. IGFs binding to their cognate receptors, IGF1R or IR-A, promote receptors phosphorylation and downstream activation of the PI3K/Akt pathway. Akt and MAPK phosphorylate EphA2 at Ser897 and promote ligand-independent activity of EphA2. The IR-A phosphorylates EphB4 at Tyr987, avoiding receptor ubiquitination and degradation, thereby maintaining EphB4 steady-state levels. mRNA expression of *EphB4* is sustained by Akt-driven signals to the nucleus but the mechanism underlying this effect is not well characterized. Biological responses critical for cancer development and progression are reported.

Major connections between the IGF system and Ephrin receptors in cancer are summarized in Figure 1.

### 6.2. Hippo Pathway

The Hippo pathway is an evolutionary conserved tumor suppressive pathway with fundamental roles in organ size, cell proliferation, regeneration and stemness [221]. The pathway has a kinase component, which has inhibitory functions, and a transcriptional module [222]. The inhibitory kinase module includes the mammalian STE20-like protein kinase 1 (MST1) and MST2 and the large tumor suppressor 1 (LATS1) and LATS2 kinases; the transcriptional module includes the transcriptional coactivators yes-associated protein (YAP), its paralogue transcriptional co-activator with a PDZ-binding motif (TAZ), and the TEA domain family members (TEAD1–TEAD4) [222]. When the Hippo pathway is on, LATS1 or LATS2 phosphorylate YAP and TAZ, and this phosphorylation leads to YAP/TAZ interaction with 14-3-3 proteins, cytoplasmic retention, and proteasomal degradation [222]. When the Hippo pathway is off, YAP and TAZ are not phosphorylated by LAT1/2 and translocate into the nucleus, where they bind to transcription factors, including TEAD1–TEAD4, thereby enabling the transcription of target genes such as *CTGF* or *Cyr61* (please refer to [222] for more details). Aberrant regulation of the Hippo pathway drives tumor development and several mechanisms of cancer progression, including metastasis, EMT, and resistance to anoikis [222]. Pharmacological approaches targeting YAP/TAZ in tumor cells have been recently developed [223]. The authophagy inhibitor verteporfin is a small molecule and the most widely used Hippo pathway inhibitor. It abrogates the interaction between YAP/TAZ and TEAD, therefore affecting their transcriptional activity [222,223]. Clinical trials are currently recruiting patients to test verteporfin activity in glioblastoma (NCT04590664) and pancreatic tumors (NCT03033225).

Much evidence supports functional interactions between the IGF system and the Hippo pathway in various cancer models [224,225,226,227]. In diffuse large B-cell lymphoma, IGF1R blockade using AG1024 or PPP or short hairpin RNA decreased the expression of the oncogenic YAP protein and enhanced MST1 expression, suggesting that the IGF1R might contribute in regulating the Hippo pathway [227]. In triple negative breast cancer, cell growth and fokal adhesion formation are triggered by an IGF1/IGF1R/FAK/Akt/YAP signaling cascade [225]. In this axis, IGF1 evoked a direct interaction between the IGF1R and FAK, activation of Akt, which decreased phosphorylation of YAP, thereby promoting YAP nuclear accumulation and expression of its canonical targets like *CTGF* and *Cyr61* [225]. Accordingly, treatment with either OSI-906, FAK inhibitor VS-4718 or verteporfin ablished *CTGF* and *Cyr61* expression, confirming that the IGF1/IGF1R axis regulates YAP target genes through FAK [225]. The crucial role of Akt in the activation of the Hippo pathway was also evidenced in hepatocellular carcinoma where a positive correlation between phospho-Akt (Ser473) and nuclear YAP was observed by immunohistochemistry in tissue samples [226]. In addition, in mouse NIH-3T3 fibroblasts and HeLa cells, wartmannin, a PI3K signaling inhibitor, leads to increased phosphorylation of YAP [226].

Published data indicated that transgenic mice with mutations in members of the Hippo pathway, including LATS1/2, develop sarcomas [228,229]. Accordingly, the Hippo pathway exherts oncogenic actions in osteosarcoma, Ewing sarcoma, synovial sarcoma and rhabdomyosarcoma, supporting the rationale for targeting this axis in these tumors. In addition, some evidence supports a functional interaction between the Hippo pathway and the IGF system in osteosarcoma and synovial sarcoma [154,188,230].

RNA-seq studies demonstrated that *YAP* as well as YAP-regulated genes, such as *Cyr61*, *THBS1*, *PAI-1* and *BIRC5*, are significantly overexpressed in tissues derived from osteosarcoma patients as compared to normal bone tissues [230]. Mechanistically, the YAP/TEAD interaction increased in vitro osteosarcoma cell proliferation and in vivo tumor growth [230]. Accordingly, the YAP inhibitors verteporfin and CA3 inhibited YAP expression, YAP-derived transcriptional activity of TEAD, in vitro viability of osteosarcoma cells and reduced in vivo growth of osteosarcoma primary bone tumor [230]. Another study demonstrated that Cyr61 enhanced metastatic progression of osteosarcoma by stimulating the expression of the *IGF1R*, thus evoking EMT [154]. Similarly, a direct correlation was observed between Cyr61 and IGF1R expression in osteosarcoma tissues analyzed by immunohistochemistry. Cyr61 depletion reduced IGF1 and IGF1R expression, both at mRNA and protein levels, and downregulated IGF1R downstream effectors. Furthermore, IGF1 stimulation or Cyr61 overexpression promoted cell motility and anchorage-independent growth, while anti-IGF1 neutralizing antibodies inhibited Cyr61 action and reduced anchorage-independent growth of Cyr61-overexpressing cells [154].

YAP/TAZ upregulation is a common feature of synovial sarcoma [188]. In vitro targeting of YAP/TAZ using specific siRNAs or verteporfin resulted in decreased cell viability and reduced expression of YAP/TAZ target genes. In vivo administration of verteporfin showed great efficacy in synovial sarcoma xenografts and PDX models, as in fact it induced a significant reduction in tumor volume. A significant percentage of patient-derived tissues showed high nuclear staining of YAP and TAZ. Importantly, activation of YAP/TAZ signals in synovial sarcoma is dependent on the pathognomonic chimeric SS18-SSX fusion protein and its ability to act as a transcriptional dysregulator. SS18-SSX upregulates IGF2, which enhanced phosphorylation of the IGF1R/Akt axis and reduced phosphorylation of LATS1, YAP and TAZ, enhancing TEAD activation and YAP/TAZ/TEAD oncogenic transcriptional program [188].

The Hippo pathway plays oncogenic roles in the development and progression of rhabdomyosarcoma. YAP/TAZ is upregulated in both alveolar and embryonal sybtypes compared to normal muscle as demonstrated by immunohistochemical analysis [224,231]. Recent evidence confirmed that verteporfin inhibited cell proliferation of both embryonal and alveolar subtypes, constituting a valuable therapeutic approach for rhabdomyosarcoma [232]. In alveolar rhabdomyosarcoma cells, YAP depletion caused growth arrest and senescence [224]. Expression of active YAP in satellite cells resulted in embryonal rhabdomyosarcoma tumor formation in mice, supporting the role of the Hippo pathway in embryonal rhabdomyosarcoma tumorigenesis [233]. Accordingly, YAP knock-down rescued in vitro differentiation and decreased in vivo tumor burden [233]. Pharmacologic inhibition of the Hippo pathway using verteporfin diminished the activity of TEAD, reduced in vitro cell growth and delayed tumor growth of alveolar rhabdomyosarcoma cells xenografts [231]. TAZ depletion in embryonal rhabdomyosarcoma cells reduced cell proliferation, anchorage-independent growth, and the expression of cancer-related genes [234]. Notably, enhanced expression of TAZ in myoblasts promoted proliferation and colony formation, suggesting putative oncogenic action [234].

Recent findings in Ewing sarcoma demonstrated that TEAD triggers cytoskeletal riorganization in cells characterized by low levels of EWS-FLI1 fusion protein [235] and this is relevant considering that there are data indicating that low EWS-FLI1 levels might promote Ewing sarcoma migration, invasion and metastasis [228]. Published data suggested that TAZ might play a relevant role in metastatic onset [235]. In clinical cases mRNA expression of *TAZ* inversely correlated with FLI1 and EWS-FLI1 target genes. Furthermore, TAZ expression was associated with adverse prognosis. Overall, the subset of Ewing sarcoma cells characterized by low levels of EWS-FLI1 have an increased metastatic potential, displaying increased expression of the YAP/TAZ/TEAD complex and upregulated expression of YAP/TAZ/TEAD target genes as compared to cells expressing high levels of EWS-FLI1. In those subsets of cells, verteporfin inhibited the formation of the YAP/TAZ/TEAD complex and the expression of genes involved in migration and cytoskeletal processes [235]. Accordingly, verteporfin decreased in vitro stress fibers and focal-adhesion formation and reduced Ewing sarcoma cell-derived in vivo lung metastases. Pharmacological inhibition of YAP/TAZ/TEAD with verteporfin in cells with low expression of EWS-FLI1 inhibited migration and invasion and reduces metastatic potential [235].

**Figure 2:**
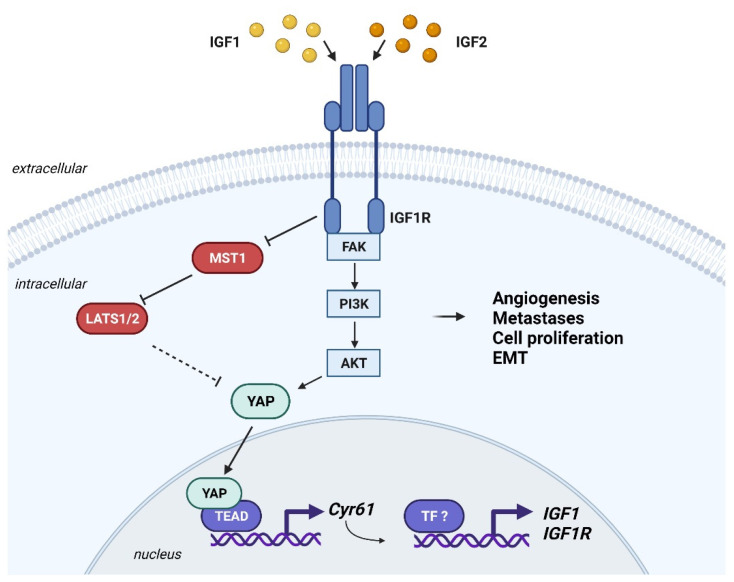
Schematic representation of the functional interaction between the IGF system and the Hippo pathway in cancer. IGF1R might contribute to regulating the Hippo pathway favoring YAP expression and translocation into the nucleus. The IGF1/IGF1R/FAK/Akt/YAP signaling cascade, which promotes YAP nuclear accumulation and expression of its canonical targets, including *Cyr61*, is depicted. Conversely, the IGF1R inhibits MST1 expression and the downstream kinase LATS1/2 thereby blocking LATS1/2-driven YAP phosphorylation and degradation. Cyr61 drives mRNA expression of *IGF1* and *IGF1R* but the mechanisms are still poorly defined. Biological responses evoked by the IGF/Hippo pathway in cancer are reported.

Major connections between the IGF system and Hippo pathway in cancer are summarized in Figure 2.

### 6.3. BET Proteins

Bromo- and extra-terminal domain (BET) proteins, such as BRD2, BRD3, BRD4, BRDT, represent a family of major epigenetic regulators connecting chromatine modifications to transcriptional activation of genes. These proteins are characterized by two tandem bromodomains that recognize acetylated-lysine residues in nucleosomal histones, thus activating transcription by recruiting the multiprotein mediator complex and positive transcription elongation factor b (P-TEFb) [236]. BET proteins play an important physiological role in homeostasis and cell survival, as in fact their dysregulation is associated with pathologic conditions including cancer [236]. BET proteins can aberrantly regulate the expression of genes involved in cell cycle (*MYC*, *CCND1*, *JUNB*), resistance to apoptosis (*BCL2*), EMT (*Snail*) and inflammatory response (*IFNγ*) [236,237]. While BRD4 is the most studied among BET proteins, there are several reports indicating that BRD2 and BRD3 might also play an important role in dysregulating oncogenes in cancer [236]. Accordingly, in recent years small molecule inhibitors were developed to block their activity. BET inhibitors competitively bind the acetyl–lysine recognition motif (bromodomain) of BET proteins leading to displacement from chromatin. Several inhibitors have been tested in clinical trials including I-BET762, PLX51107, or OTX015 and TEN-010, which are structurally similar to the most widely used JQ1 [236].

There is evidence in the literature connecting BET protein action and the IGF system in cancer [238,239,240,241,242]. *IGF1R* and *INSR* genes are direct targets of BRD4 in several cancer models [240]. In particular, ChIP-seq data for BRD4 binding demonstrated enrichment of BRD4 occupancy at the transcriptional start site (TSS) of the *IGF1R* and *INSR* genes in HeLa, HEK293T as well as MM.1S cells, a multiple myeloma cell line [240]. Treatment of RKO-1, PC-3, and MCCL-357 cells with BET inhibitors JQ1 or MS417 reduced BRD4 binding to the *IGF1R* and *INSR* TSS, downregulated their mRNA expression and inhibited the PI3K pathway and Akt activation [240]. Accordingly, dual PI3K/BET inhibition blocked the PI3K/Akt pathway by eliminating the feedback loop reactivating the PI3K pathway in the presence of the PI3K single agent, attenuated cell growth in multiple in vitro tumor models and displayed in vivo antitumor action [240]. The efficacy of BET and PI3K inhibitors was demonstrated in alveolar and embryonal rhabdomyosarcoma cell lines where the combination of JQ1 and the PI3K inhibitor BYL719 gave synergistic effects in rhabdomyosarcoma cells causing G1 arrest and caspase-dependent apoptosis [241]. Similarly, in osteosarcoma cells, JQ1 inhibited proliferation and survival, inducing G1 arrest and in vitro senescence. Other evidence indicated that JQ1 as monotherapy had no effect on the size of MNNG/HOS tumor xenografts [242]. On the contrary, the combined treatment with JQ1 and the mTOR inhibitor rapamycin synergistically inhibited growth and survival of osteosarcoma cells both in vitro and in vivo experiments [242].

A rationale for co-targeting the IGF1R/Akt axis and BET proteins was provided in Ewing sarcoma where the expression of a constitutively active Akt significantly decreased sensitivity of Ewing sarcoma cells to the BET inhibitor NHWD870, suggesting a crucial role of the IGF1R/Akt cascade in resistance of Ewing sarcoma cells to BET inhibitors [239]. Accordingly, the combined treatment with the IGF1R kinase inhibitor BMS754807 and NHWD870 gave synergistic effects in vitro and induced tumor regression in Ewing sarcoma cell line-derived xenograft tumors [239]. This effect was likely independent of any action in modulating EWS-FLI1 levels [239], even though other evidence demonstrated the ability of BET inhibitors to suppress EWS-FLI1-dependent transcription and downregulation of target genes like *IGF1* [238]. As recently shown, EWS/FLI1 is part of a large complex including BRD4, RNA Pol II and mediator complex 1, supporting the notion that EWS/FLI1 requires BET proteins, particularly BRD4, for its transcriptional program [243]. Accordingly, BRD4 knockdown or JQ1 treatment attenuated EWS/FLI1-modulated transcriptional signature [243]. BET inhibitors inhibited autocrine IGF1 action and activation of the IGF1R/Akt pathway in Ewing sarcoma cells [238]. Accordingly, BET inhibitors impaired cell viability, clonogenic survival of Ewing sarcoma cells and blocked EWS-FLI1-induced transformation of mouse NIH3T3 fibroblasts [238].

The BET inhibitor JQ1 inhibited mRNA and protein expression of the RNA-binding protein IGF2BP3 in Ewing sarcoma and B cell acute lymphoblastic leukemia [244,245]. Whether IGF2BP3 might be a direct target of BET proteins and the molecular mechanism underlying this effect is not known at the moment but there is some evidence in Ewing sarcoma cells indicating that silencing of IGF2BP3 conferred resistance to JQ1, thereby supporting a connection between BET proteins and IGF2BP3 [244]. Notably, IGF2BP3 sustained *IGF2* and *IGF1R* mRNA translation in tumor cells [10] but any involvement of the IGF2BP3/IGF2/IGF1R axis in response to BET inhibitors has not been yet established.

**Figure 3:**
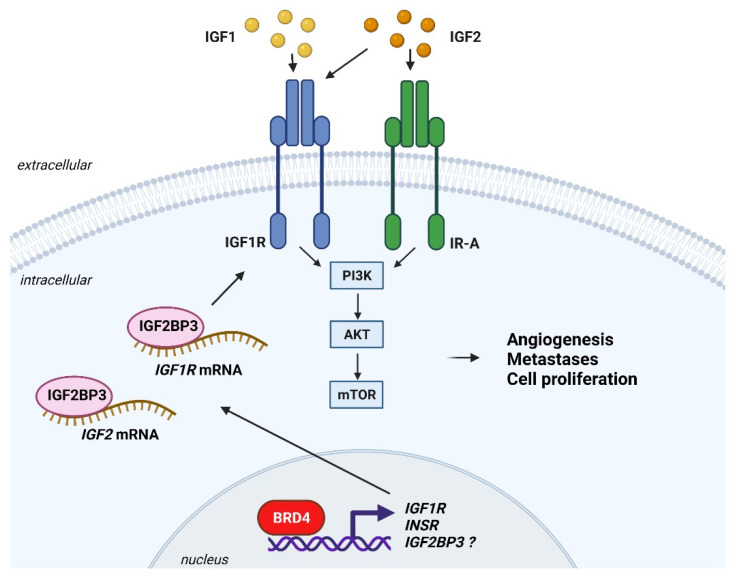
Schematic representation of the functional cross-talk between the IGF system and the BET proteins in cancer. In the nucleus, BRD4 binds the transcriptional start site of the *IGF1R* and *INSR* and modulates their expression. Whether BRD4 might directly regulate *IGF2BP3* mRNA expression has to be established. In the cytoplasm, IGF2BP3 sustains *IGF2* and *IGF1R* translation. Ligand binding to the IGF1R or IR-A activates the PI3K/Akt/mTOR. Biological responses evoked by the IGF/BET proteins in cancer are reported.

Major connections between the IGF system and the BET proteins in cancer are summarized in Figure 3.

### 6.4. CXCR4

The G protein-coupled receptor chemokine (C-X-C motif) receptor 4 (CXCR4) is the membrane receptor for the chemokine CXCL12 and was originally identified in peripheral blood leukocytes and other cell types like hematopoietic stem cells, stromal fibroblasts and tumor cells [246,247]. Ligand binding leads to the release of G protein subunits, which bind and activate phospholipase C, PI3K/Akt or Ras/MEK/ERK signaling pathways. In cancer, CXCR4-dependent signaling mediates various processes including cell survival, proliferation, migration, adhesion, stress resistance [247]. Various CXCR4 antagonist have been developed and tested in clinical trials. An active phase IIb trial is currently testing the efficacy of the CXCR4 antagonist BL-8040 in combination with pembrolizumab, in patients with pancreatic cancer (NCT02907099). A phase IIa trial is testing the safety profile, tolerability and pharmacodynamics effects of BL-8040 in combination with nelarabine for relapsed or refractory T-acute lymphoblastic leukemia/lymphoblastic lymphoma (NCT02763384). A phase I study will investigate the safety, tolerance and efficacy of CXCR4 modified CAR T cells in patients who failed standard treatment/refractory/relapsed multiple myeloma (NCT04727008) while the CXCR4 antagonist AMD3100 was tested in a variety of tumor types [247].

Crosstalk between the CXCR4 and IGF axis has been documented in embryonal development and cancer [247,248]. In embryonic germline stem cells, a reciprocal transactivation was observed between the IGF1/IGF1R and CXCL12/CXCR4 axes [248]. Indeed, IGF1 stimulated IGF1R, Akt and CXCR4 phosphorylation, which was suppressed by PPP. In addition, CXCL12 or AMD3100 treatments effectively increased or suppressed the phosphorylation of CXCR4, Akt, and IGF1R, respectively. Mechanistically, both IGF1 and CXCL12 increased cell migration while PPP inhibited the effect of CXCL12 on cell migration [248]. A functional interaction between the IGF1R and CXCR4 was documented in breast cancer cell lines [249] where CXCR4 and IGF1R directly interacted at the cell membrane, thereby inducing IGF1-evoked CXCR4 transactivation and cell migration independently of its cognate ligand CXCL12 [249].

CXCR4 plays an important role in tumor progression and metastases of several sarcoma subtypes [246]. Evidence demonstrating the oncogenic role of CXCR4 and a putative role as therapeutic target is present in Ewing sarcoma, osteosarcoma, rhabdomyosarcoma and synovial sarcoma. In Ewing sarcoma, the expression of CXCR4 is highly dynamic as it is transiently induced by microenvironmental stress such as hypoxia or serum-deprivation [250]. Notably, the subpopulation of Ewing sarcoma cells characterized by high CXCR4 expression display high migratory capabilities [250]. Accordingly, targeting the CXCL12/CXCR4 axis inhibited the metastatic capability of Ewing sarcoma cells [250]. As recently demonstrated, the RNA-binding protein IGF2BP3 controlled CXCR4 expression levels through post-transcriptional regulation of its functional partner CD164, thus mediating cell migration toward CXCL12 in hypoxic conditions [251]. Taking into account that IGF2BP3 sustains IGF1R expression as well [162], we can speculate that co-targeting the IGF axis and CXCR4 in Ewing sarcoma cells expressing high levels of IGF2BP3 might represent a valuable therapeutic option.

In synovial sarcoma, CXCR4 mRNA and protein expression levels are upregulated in tumor as compared to non-tumor tissues [252]. In addition, CXCR4 levels were slightly higher in tissues from patients presenting metastases compared to tissues from non-metastatic patients [252]. Interestingly, positive CXCR4 nuclear staining was significantly associated with poor survival in a cohort of 80 synovial sarcoma cases [187]. The authors also explored nuclear co-expression of CXCR4 and IGF1R and CXCR4/IGF1R/nuclear double positive staining was associated with poorer survival compared to CXCR4 or IGF1R alone [187]. This observation is just correlative at the moment but supports the rationale for further exploring the functional CXCR4–IGF1R interaction and its clinical relevance in synovial sarcoma.

In osteosarcoma cases, high levels of CXCR4 mRNA and protein expression were correlated with tumor progression, metastatic disease, poor metastasis-free and overall survival [253]. Anti-CXCR4 agents including AMD3100 and the fully human CXCR4 antibody MDX1338 determined a slight decrease in cell proliferation and migration, supporting the use of these compounds as combination therapy [253]. A direct connection between CXCR4 and the IGF system has not been demonstrated so far in osteosarcoma but downstream effectors of RTK affect CXCR4 expression in this tumor. Accordingly, recent findings have uncovered a relationship between PTEN/Akt and CXCR4 signaling pathways in osteosarcoma [254]. While the CXCL12/CXCR4 signaling stimulates Akt phosphorylation, the use of the Akt inhibitor AKTi-1/2 decreased CXCL12 and CXCR4 mRNA levels in human 143B and murine MOTO osteosarcoma cell lines [254]. Interestingly, loss of PTEN using siRNA or the PTEN inhibitor VO-OHpic activated Akt and increased *CXCL12* and *CXCR4* mRNA levels [254]. Collectively, these data suggest the existence of a positive feedback CXCR4–Akt–CXCR4 loop, which sustains CXCR4 expression, and which is antagonized by the antitumor effect of PTEN. From the functional standpoint, the PTEN/Akt/CXCR4 nexus enhanced in vitro cell proliferation and migration while inhibiting apoptosis [254]. In vivo, NOD/SCID mice injected with 143B cells stably transfected for the forced overexpression of PTEN displayed reduced bone destruction, mitigated tumor burden, decreased CXCR4 mRNA and protein levels (as evaluated by quantitative PCR and immunohistochemistry in tumors harvested from mice), and reduced metastatic tumor growth in the lungs, compared to control mice [254].

In rhabdomyosarcoma, the CXCL12/CXCR4 axis is crucial for the metastatic process and high CXCR4 expression is associated with poor outcome [255]. CXCR4 overexpression significantly enhanced cell motility, effect that was more robust in alveolar vs. embryonal rhabdomyosarcoma subtypes [255]. Accordingly, anti-CXCR4 blocking antibody (MDX1338) efficiently reduced migration and invasion of alveolar rhabdomyosarcoma RH30 cells [256]. Interestingly, previous studies in rhabdomyosarcoma cells demonstrated that CXCR4 overexpression was sustained by the upregulation of Sp transcription factors (80% of cases) [257]. Sp knockdown in RD and RH30 cells decreased the expression of *CXCR4* as well as other major SP-target genes including the *IGF1R*, *c-met* and *PDGFRα*, which might represent putative targets for combinatorial treatments [257].

**Figure 4:**
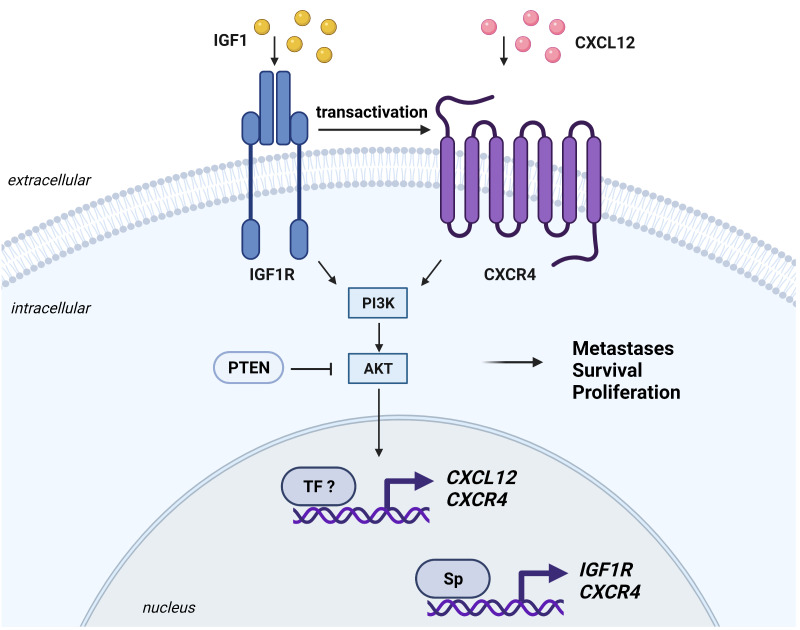
Schematic representation of the functional connection between the IGF system and the CXCR4 signaling in cancer. At the cell membrane, the IGF1/IGF1R axis evokes CXCR4 transactivation, independently of its cognate ligand CXCL12. In the cytoplasm, both IGF1R and CXCR4 stimulate Akt phosphorylation, which is inhibited by PTEN. In the nucleus, Akt transmits signals that favor *CXCR4* and *CXCL12* mRNA expression, but the exact mechanism is still poorly understood. Sp transcription factors sustain mRNA expression of both the *IGF1R* and *CXCR4*. Biological responses evoked by the IGF/CXCR4 pathways in cancer are reported.

Major connections between the IGF system and CXCR4 signaling in cancer are summarized in Figure 4.

## 7. Conclusions

Dysregulation of the IGF system drives cancer cell proliferation, migration, EMT, and drug resistance. Accordingly, multiple anti-IGF therapeutic strategies have been developed to block this pathway, including IGF1R monoclonal antibodies, tyrosine-kinase inhibitors, IGFs neutralizing antibodies or the new IGF-TRAP and IGF1R—targeted antibody—drug conjugates. After decades of research in the field, clinical relevance of therapeutic agents targeting the IGF system in cancer appears limited to subsets of sarcomas. This is in line with the exceptional dependence of these diseases on IGF-driven processes. Still, even in those tumors, molecular profiling to identify patients who would better respond to anti-IGF-based treatment is still an urgent clinical need. In addition, any therapeutic strategy solely targeting the IGF axis is likely insufficient to block tumor growth. The majority of the information obtained to date shows the complexity of the IGF axis regulation and the multiplicity of interactions with other cancer-relevant pathways, which either potentiate or compensate molecular signaling mediated by the IGF system. A better understanding of this network of signaling pathways might contribute to the identification of novel and more effective therapeutic combinations for sarcoma treatment.
